# Infections with the Microbe *Cardinium* in the Dolichopodidae and Other Empidoidea

**DOI:** 10.1673/031.013.4701

**Published:** 2013-06-04

**Authors:** Oliver Y. Martin, Nalini Puniamoorthy, Andrea Gubler, Corinne Wimmer, Christoph Germann, Marco V. Bernasconi

**Affiliations:** 1 ETH Zürich, Experimental Ecology, Institute for Integrative Biology, CHNJ 11, Universitätsstrasse 16, CH-8092 Zürich, Switzerland; 2 Institute of Evolutionary Biology and Environmental Studies, University of Zurich, Winterthurerstrasse 190, CH8057 Zürich, Switzerland; 3 Department of Biological Sciences, National University of Singapore, 14 Science Drive 4, Singapore I 17543, Singapore; 4 Natur-Museum Luzern, Kasernenplatz 6, CH-6003 Luzern, Switzerland; + these authors contributed equally

**Keywords:** reproductive parasite, *Rickettsia*, *Spiroplasma*, symbiont, *Wolbachia*

## Abstract

Maternally transmitted reproductive parasites such as *Wolbachia* and *Cardinium* can drastically reshape reproduction in their hosts. Beyond skewing sex ratios towards females, these microbes can also cause cytoplasmic incompatibility. *Wolbachia* probably infects two thirds of insects, but far less is known about the occurrence or action of other bacteria with potentially similar effects. In contrast with the two more widespread reproductive parasites, *Wolbachia* and *Spiroplasma*, far less is known of infections with *Cardinium* (Bacteroidetes) and possible consequences in the Diptera. Here, in an extensive survey, 244 dipteran species from 67 genera belonging to the Dolichopodidae, Empididae, and Hybotidae were assessed for the presence of the microbe *Cardinium*. Although 130 of the species screened tested positive (ca. 53%), the presence of *Cardinium* could only be confirmed in 10 species (ca. 4%) based on analysis of sequences. Numerous additional sequences were found to be assignable to known or unknown Bacteroidetes.

Considering the known issues concerning specificity of *Cardinium* primers and the phylogenetic uncertainties surrounding this microbe, the actual prevalence of this symbiont is worthy of further scrutiny. Potential directions for future research on *Cardinium-host* interactions in Diptera and in general are discussed.

## Introduction

Maternally inherited reproductive parasites such as *Wolbachia, Rickettsia*, and *Spiroplasma* species are known to have profound effects on reproduction and behavior (e.g., Goodacre et al. 2009) of their hosts (reviewed in Engelstädter and Hurst 2008; Goodacre and Martin 2012). More recently, a further reproductive parasite, *Cardinium hertigii* (Bacteroidetes) (Zchori-Fein et al. 2004), has been described. *Cardinium* infections were first observed in ticks (Kurtti et al. 1996), and are found in a wide range of spiders, mites, and other arachnids (Zchori-Fein and Perlman 2004; Chigira and Miura 2005; Groot and Breeuwer 2006; Enigl and Schausberger 2007; Duron et al. 2008b; Gruwell et al. 2009; Martin and Goodacre 2009; Chang et al. 2010; Breeuwer et al. 2012). Although this microbe seems to be more common in arachnids, it is also known to infect insects (Zchori-Fein and Perlman 2004; Provencher et al. 2005; Bigliardi et al. 2006; Marzorati et al. 2006; Sirviö and Pamilo 2010).

*Cardinium* is now known to cause three of the four classic phenotypes often associated with reproductive parasites: cytoplasmic incompatibility in the parasitoid wasp *Encarsia pergendiella* (Hunter et al. 2003), the spider mite *Bryobia sarothamni* (Ros and Breeuwer 2009), and the tetranychid mites *Eotetranychus suginamensis* (Gotoh et al. 2006) and *Tetranychus cinnabarinus* (Xie et al. 2010); feminization in *Brevipalpus* mites (Weeks et al. 2001); and parthenogenesis in the hemipteran *Aspidiotus nerii* (Provencher et al. 2005) and in *Encarsia* species (Zchori-Fein et al. 2001; 2004). In contrast to the better-known symbionts *Wolbachia* and *Spiroplasma*, the parasite *Cardinium* has so far not been found to be associated with male-killing.

Surveys for infections with the two more common reproductive parasites, *Wolbachia* and *Spiroplasma*, are available for many arthropods (e.g., for spiders: Goodacre et al. 2006). Within the Diptera, previous extensive surveys have focused on the genus *Drosophila* (Clark et al. 2005; Mateos et al. 2006), the Empidoidea (Martin et al. 2013), and the Muscoidea, including the yellow dung fly, *Scathophaga stercoraria* (Martin et al. 2012). Both these symbionts have been shown to manipulate host reproduction in flies. *Wolbachia* causes cytoplasmic incompatibility in *Culex pipiens* (Yen and Barr 1971), and *Spiroplasma* is associated with male-killing in *Drosophila willistoni* (Hackett et al. 1986). *Wolbachia* has also been shown to affect other reproductive traits in *Drosophila*. Specifically, the parasite increases male mating rate (Champion de Crespigny et al. 2006) and decreases sperm competitive ability (Champion de Crespigny and Wedell 2006). However, knowledge of infections with *Cardinium* and possible consequences in the Diptera lags far behind.

As outlined above, Diptera, and especially *Drosophila* flies, have been instrumental in furthering the understanding of hostreproductive parasite interactions. With over 7,100 species worldwide, the Dolichopodidae, long-legged flies, represent one of the most speciose families in the Diptera (Pape et al. 2009). The actual number of taxa is presumably higher, especially in the tropics, where dozens of new taxa are discovered every year (e.g., Bickel 2009). The greatest diversity and abundance of long-legged flies are found at humid sites, and the flies show high potential as bio-indicators for natural quality assessments of biotopes due to their specific habitat requirements (Pollet 2009). Adult and larval stages of most species feed on small soft-bodied arthropods (Ulrich 2004), and because of this they are considered to be of potential use in biocontrol. Finally, their elaborate courtship behavior and their conspicuous male secondary sexual characters further provide excellent opportunities for studying sexual selection and speciation processes (Germann et al. 2010; Martin et al. 2013). This is particularly relevant, as reproductive parasites have been shown to have profound effects on reproductive traits and potential involvement in reproductive isolation (Wade and Stevens 1985), with obvious direct links to the study of sexual selection (discussed in Martin et al. 2012; 2013).

Considering the dramatic effects the microbe *Cardinium* can have on its arthropod hosts, it is important to acquire further knowledge, both concerning the range of hosts affected and the precise consequences of infection. Moreover, it has been suggested that a greater range of arthropods should be screened in order to achieve a better understanding of *Cardinium* phylogenetic diversity (Nakamura et al. 2009). Surprisingly, knowledge of *Cardinium* infections within the Diptera remains particularly hazy. Here, 244 fly species from 67 genera belonging to the Dolichopodidae, Empididae, and Hybotidae (all superfamily Empidoidea) were surveyed for the presence of this reproductive parasite (as also done for *Wolbachia, Spiroplasma*, and *Rickettsia* in Martin et al. 2013).

## Methods

### Samples, DNA extraction, amplification

Extensive DNA samples from previous studies (Bernasconi et al. 2007a; 2007b; Germann et al. 2010; [Bibr bibr19][Bibr bibr20]; Pollet et al. 2010; 2011) and a few additional fly samples were used to assess a range of representatives of the Dolichopodidae and species from other empidoidfamilies, namely Empididae and Hybotidae, for infection with *Cardinium* via PCR (see Martin et al. 2013 for data on three other symbionts). DNA was extracted from whole fly specimens using DNeasy Tissue kits (Qiagen AG, www.qiagen.com) according to the manufacturer's instructions. Whole specimens were triturated mechanically in microtubes using a TissueLyser (Mixer Mill MM 300, Qiagen AG). Following digestion with Proteinase K (2 µg/mL), samples were applied to the columns for DNA absorption and washing. Finally, DNA was eluted in 200 µl of the buffer from the kit and stored at -20° C. All the extracted fly specimens were deposited at the Zoological Museum, Institute of Evolutionary Biology and Environmental Studies, University of Zurich. Standard PCR reactions were performed with 2 µl of the extracted DNA as template, 1 µl of each primer (10 µM), 12.5 µl Master Mix (250 units, HotStarTaq Master Mix Kit, Qiagen AG), and 8.5 µl distilled H_2_O, for a total volume of 25 µl (manufacturer's buffer). The following specific primers (Microsynth GmbH, www.microsynth.ch) were used: *Cardinium* (16S rDNA gene), CardiniumCh-F: TACTGTAAGAATAAGCACCGGC, and CardiniumCh-R:GTGGATCACTTAACGCTTTCG (ZchoriFein and Perlman 2004). The PCR reaction mixtures were subjected to 10 min DNA denaturation at 95° C, 50 cycles of denaturation at 94° C for 30 sec, annealing at 50° C for 20 sec, and elongation at 72° C for 30 sec. Elongation was completed by a further 7 min step at 72° C. PCR reactions were performed in a DNA Thermal Cycler (Perkin-Elmer Applied Biosystems, www.perkinelmer.com). Purification of PCR products for direct sequencing was performed by adding 0.5 µl (1 U/µl) Shrimp Alkaline Phosphatase (Promega AG, www.promega.com), 0.25 µl (20 U/µl) Exonculease I (New England Biolabs (Bioconcept), www.neb.com), and 24.25 µl distilled H_2_O (ratio of PCR product and ExoSap-mix 1:1) to each PCR product. The ExoSAP protocol consisted of 45 min incubation at 37° C and 15 min deactivation at 80° C. Cycle sequencing reactions were performed in total volumes of 10 µl using an ABI Prism Big Dye Terminator Cycle Sequencing Kit (Perkin-Elmer Applied Biosystems) on an ABI 3730 DNA Analyser (Perkin-Elmer Applied Biosystems), again following the manufacturer's instructions.

Negative and positive controls were used during the amplification procedures. The negative controls consisted of micro-tubes/positions in the reaction plates containing all the necessary reagents, except that the extracted genomic DNA to be amplified was substituted with distilled H_2_O. Positive controls consisted of extracted genomic DNA of samples infected with the microorganism under study (for the PCR) or in purified PCR products (for the direct sequence). Results of PCR amplification were visualized via Gel Electrophoresis.

### DNA sequence analyses

Gene sequences were handled and stored using the Lasergene program Editseq (DNAstar Inc., www.dnastar.com). Sequence alignment was performed using the Clustal W method as implemented in Megalign (DNAstar Inc.) using the default multiple alignment parameters (gap penalty =15; gap length penalty = 6.66; delay divergent sqs (%) = 30; DNA transition weight = 0.50). All obtained microbial sequences were checked in GenBank using the BLAST tool to verify their identity, i.e., to determine their similarity to known *Cardinium* sequences or, conversely, to find out their possible identity as other Bacteroidetes or unknown (taxonomically undescribed) bacteria.

## Results

Out of the 244 species screened, over half of the species tested positive (130/244, ca. 53%; see results of PCR survey displayed in Table 1), with positives found across all three families surveyed. At the genus level, the following patterns were observed: in the Empididae, five out of seven genera (ca. 71%) tested positive, compared with 4/9 (ca. 44%) in the Hybotidae and 35/51 (ca. 69%) in the Dolichopodidae (*sensu lato + sensu stricto; see* Pollet and Brooks 2008). However, subsequent sequencing of amplicons, and searching for most closely related bacterial sequences, indicated that not all of these positives can be assigned to *Cardinium*. Of the 169 sequences amplified, 15 had to be discarded because the quality was too poor to allow analysis. Of the remaining 154, only 10 of the sequences came out as clearly assignable to *Cardinium* (*see* overview in Table 2). These sequences stem from species from five subfamilies within the Dolichopodidae *s. stricto* representing the genera *Argyra, Chrysotus, Medetera, Peleropeodes, Sciapus*, and *Tachytrechus*. In six cases, the closest matches were sequences obtained from three hemipterans (Table 2). The other sequences matched either the hymenopteran *Plagiomerus diaspidis*, the copepod *Nitocra spinipes*, or the spider mite *Tetranychus cinnabarinus* (Table 2).

A further 50 sequences could be assigned to other Bacteroidetes as either known or unknown microbes (N = 27) (Table 2). The remaining sequences belonged to the Proteobacteria, including 13 matching the opportunistic pathogen *Pseudomonas (see* D'Argenio et al. 2001). A sequence obtained from one *Dolichopusplumipes* sample closely matched the pathogen *Rickettsiella tipulae* (Gammaproteobacteria), known to infect the crane fly, *Tipula paludosa* (Leclerque andKleespies 2008; Bouchon et al. 2012). The remaining sequences were either unknown Proteobacteria (N = 28) or unknown bacteria (N = 38).

## Discussion

The species from the Dolichopodidae, Empididae, and Hybotidae assessed here seemed to show a high proportion of infections based on PCR results alone. This would be noteworthy, as *Cardinium* is generally thought to be less widespread than other symbionts such as *Wolbachia* and *Spiroplasma*. Whereas *Wolbachia* is thought to infect ca. 66% of insects (Hilgenboeker et al. 2008), previous surveys indicated that *Cardinium* may be much rarer overall in arthropods and are perhaps restricted to particular groups, such as Hemiptera (see Nakamura et al. 2009). For example, extensive surveys across spiders and other arachnids indicated that proportions of infected samples were generally higher than in insects (Weeks et al. 2003; Duron et al. 2008b; Martin and Goodacre 2009). Nevertheless, Zchori-Fein and Perlman (2004) included 85 insect species in their study, and found that only 6% of species screened tested positive for *Cardinium* versus 24% positive for *Wolbachia*. This low prevalence in insects was mirrored in our study, as a similar prevalence of ca. 4% (10/244) was found using only those infections that were confirmed via direct sequencing.

Concerning the more specific situation in Diptera, some fly species have been assessed as part of more general surveys encompassing various arthropod groups. Zchori-Fein and Perlman (2004) assessed 11 species of Diptera as part of their survey: *Lucilia ciricata* (Calliphoridae); *Asphondylia* capparis,*Dasineuriola* sp., and *Schizomyia* sp. (Cecidomyiidae); *Culicoides circumscriptus* and *C. imicola* (Ceratopogonidae); *Culex pipiens* (Culicidae); *Drosophila melanogaster* and *D. simulans* (Drosophilidae); *Musca domestica* (Muscidae); and *Ceratitis capitata* (Tephritidae). *C. pipiens* and the two species of *Drosophila* were found to be infected with *Wolbachia*, but none of the species assessed were infected with *Cardinium*. Similarly, no infections with *Cardinium* were found in a survey of 181 strains representing 35 species of *Drosophila* (Mateos et al. 2006). Duron et al. (2008) also surveyed 25 dipteran species as part of a larger survey and, again, found no evidence for infections with *Cardinium*. Finally, Nakamura et al. (2009) assessed *Culicoides* biting midges and described infections with a new *Cardinium* group in four out of 25 species tested. Here, the first extensive survey specifically assessing possible infections with *Cardinium* in a wider range of fly species is provided, and the presence of *Cardinium* in 10 species from six genera is confirmed. So far, to our knowledge, our survey represents the only evidence for infections with *Cardinium* in Diptera other than *Culicoides* (Nakamura et al. 2009).

The majority of sequences could not be assigned to *Cardinium*, although numerous sequences were assignable to other Bacteroidetes within the survey. Some of these sequences may well represent *Cardinium* infections, so this possibility definitely warrants further scrutiny. However, it must also be noted that the situation is complicated by the fact that the phylogenetic relationships within *Cardinium* are still confused and hence remain fluid (see Gruwell et al. 2009; Nakamura et al. 2009; Perlman et al. 2010; discussed in Breeuwer et al. 2012). To a certain extent, this problem also applies to reproductive parasites in general; for example, whether the far more intensively studied *Wolbachia* represents one or two species is still debated (Lo et al. 2007; Pfarr et al. 2007). This problem may be additionally compounded by general issues of false negatives inherent to PCR-based screening (see Breeuwer et al. 2012), so overall, it is likely that frequency of infection with symbionts is being underestimated and novel bacteria are being missed (see e.g., Weinert et al. 2007). In our study, the Ch primer set (Zchori-Fein and Perlman 2004) was used, as this primer pair is commonly used for detection of *Cardinium* (Breeuwer et al. 2012). Further work would be needed to resolve the issue, and one option would be to use more than one primer pair, or supplement molecular data with morphological analysis, as done by Nakamura et al. (2009). If some of these sequences are in fact *Cardinium*, the microbe may be a more widespread symbiont of arthropods than previously assumed.

Unsurprisingly, considering the glaring paucity of knowledge concerning the range of Dipteran species infected, so far nothing at all is known of the consequences of infection for hosts in this group. Now that it has been established that *Cardinium* can infect additional dipteran hosts from an important group in the context of sexual selection, it would be worthwhile to investigate potential consequences. As noted above, *Cardinium* can cause sex ratio distortion or reproductive alterations in its hosts. So far, research has demonstrated association with parthenogenesis in Hemiptera, parthenogenesis and cytoplasmic incompatibility in Hymenoptera, and cytoplasmic incompatibility and feminization in Acarida (reviewed in Duron et al. 2008a). Effects need not be so drastic though, as *Cardinium* has also been shown to be associated with increased fecundity in the predatory mite *Metaseiulus occidentalis* (Weeks and Stouthamer 2004). It would be highly interesting to assess the effects *Cardinium* has on its Dipteran (and other, e.g., spider) hosts.

Based on work on other symbionts, the consequences of infections can be extraordinarily varied and affect not only reproduction, but also immunity or non-reproductive behaviours. For example, *Wolbachia* infections can increase resistance to viruses in *Aedes aegypti* (Bian et al. 2010), and *Rickettsia* infection can hamper long-range dispersal behavior in a spider (Goodacre et al. 2009). Another area worthy of investigation is what happens when hosts harbor multiple infections (see Table 2). Co-infections with different strains of the same bacterium or different reproductive parasites are known to occur across arthropods (Weeks et al. 2003; Zchori-Fein and Perlman 2004; Goodacre et al. 2006; Gotoh et al. 2006; Duron et al. 2008a; Skaljac et al. 2010; Goodacre and Martin 2012). This problem has been the focus of theoretical (e.g., Engelstädter et al. 2008; Vautrin et al. 2008) and empirical study focusing specifically on interactions between *Cardinium* and the widespread *Wolbachia* (Gotoh et al. 2006; Ros and Breeuwer 2009; White et al. 2009; Sirviö and Pamilo 2010).

In conclusion, this is the first extensive survey specifically assessing possible infections with *Cardinium* in a wide selection of Diptera belonging to the empidoid families Dolichopodidae, Empididae, and Hybotidae (see Martin et al. 2013 for other symbionts). Although a large number of species tested positive for *Cardinium* in the PCR survey, only 10 of the 154 sequences analyzed could be assigned with certainty to *Cardinium*. These sequences stem from 10 species representing five subfamilies within the Dolichopodidae. We suggest that both the issues surrounding specificity of *Cardinium* primers (see Breeuwer et al. 2012) and the related question of how commonly this microbe might really infect arthopods *in toto* require further resolution. Finally, little is known of the effects this microbe has in Diptera, so possible impacts on its hosts and interactions with other endosymbionts in this speciose group are worthy of future study.

**Table 1.**
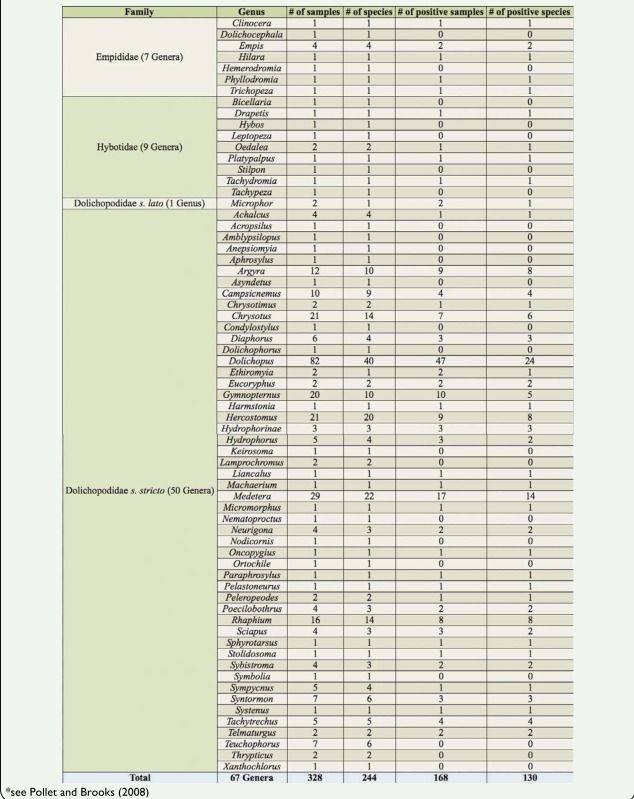
Overview of the number of infected species and genera as determined via PCR screens.

**Table 2.**
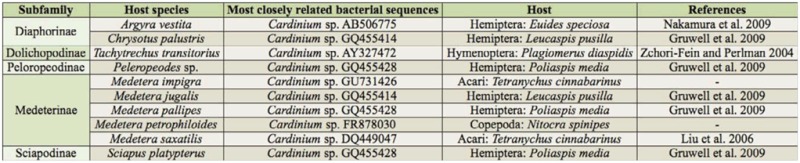
Overview of *Cardinium* sequences. Evidence for multiple infections when present data is combined with Martin et al. 2013. *M. impigra* and *M. petrophiloides* samples were also infected with *Spiroplasma, M. saxatilis* and *Sciapus platypterus* additionally harbored *Wolbachia*, whereas *A. vestita* and *Chrysotus palustris* were infected with both *Spiroplasma* and *Rickettsia*.
